# Pharmacy Students’ Attitudes Toward Distance Learning After the COVID-19 Pandemic: Cross-Sectional Study From Saudi Arabia

**DOI:** 10.2196/54500

**Published:** 2024-03-15

**Authors:** Saud Alsahali, Salman Almutairi, Salem Almutairi, Saleh Almofadhi, Mohammed Anaam, Mohammed Alshammari, Suhaj Abdulsalim, Yasser Almogbel

**Affiliations:** 1 Department of Pharmacy Practice College of Pharmacy, Qassim University Buraydah Saudi Arabia

**Keywords:** distance learning, e-learning, pharmacy education, team based learning, educational experience.

## Abstract

**Background:**

Electronic learning refers to the use of assistive tools in offline and distance learning environments. It allows students to access learning tools and materials anytime and anywhere. However, distance learning courses depend on several factors that affect the quality of learning, which consequently affect students’ preferences in the settings and tools used to deliver educational materials.

**Objective:**

This study aimed to evaluate students’ preferences for continuing distance learning after the pandemic and to assess the distance educational environment after the pandemic. It also aimed to identify the factors affecting distance learning and evaluate students’ preferences regarding modes of communication with instructors.

**Methods:**

A web-based survey was used to conduct this cross-sectional study. The target participants of this study were students in the doctor of pharmacy program at Unaizah College of Pharmacy, Qassim, Saudi Arabia. All students enrolled from December 2022 to January 2023 received an invitation with a link to the web-based survey.

**Results:**

The survey was completed by 141 students (58 female students and 83 male students). The research results showed that most students (102/141, 72.3%) did not wish to continue distance education for laboratory courses, and 60.3% (85/141) did not wish to continue taking distance team-based learning after the pandemic. Additionally, 83.7% (118/141) of the students indicated that distance courses were simple. More than half of the participants (79/141, 56%) stated that having a camera on during class negatively impacted their learning, and only 29.1% (41/141) of the students stated that nonvisual communication with their fellow students impacted their learning. A large proportion of students (83/141, 58.9%) reported impairment of social engagement on campus, 44% (62/141) in-person interactions during classes, and 73.7% (104/141) were relieved that their classes were not disrupted.

**Conclusions:**

Similar to all types of education, distance learning is characterized by advantages and disadvantages, as reported by students. Students felt that the course material was intelligible, and the distance course was uncomplicated. Moreover, they expressed relief that their studies were not disrupted. However, they also reported the loss of face-to-face contact during courses as the most significant drawback of distance learning versus face-to-face learning, followed by a lack of social connection on campus.

## Introduction

In December 2019, unexpected cases of severe respiratory illness were found in Wuhan, the capital city of China’s Hubei province [1]. The World Health Organization recognized a new coronavirus, termed SARS-CoV-2, which caused the outbreak of COVID-19 [2]. The initial COVID-19 instance in Saudi Arabia was detected on March 3, 2020. Consequently, Saudi Arabia placed most of its public and private facilities under lockdown and instituted statewide limits on population movement. Due to the widespread mitigation efforts implemented by several nations, the COVID-19 pandemic disrupted the daily lives of millions of people [3]. On March 8, 2020, the Saudi Arabian government announced a conversion from regular classroom education to distance education. In the following days, all Saudi Arabian colleges and institutions implemented this approach. Saudi Arabia was among the first nations in the Middle East to enforce full quarantine and the urgent switch to distance learning [4].

The use of assistive tools in both offline and distance learning environments is referred to as e-learning [5] and includes synchronous and asynchronous modes. Synchronous distance learning permits live interactions between educators and students through chats and video conferences, while asynchronous distance learning is based on emails and recorded videos [6]. Distance learning can be used in place of, or in addition to, traditional education [5]. Before the pandemic, some colleges had used distance education using learning management systems such as Blackboard (Anthology Inc) and Zoom (Zoom Video Communications, Qumu Corporation). Distance education delivery abruptly and entirely replaced traditional classroom education, resulting in problems such as incomplete examinations and difficulties with courses requiring hands-on training or laboratory activities [4]. After the COVID-19 outbreak forced the closure of universities in Saudi Arabia, the Saudi Ministry of Education created an alternative policy to support distance education and provided public universities with resources to aid distance learning and virtual classrooms. This was done to prevent interruptions in the educational process. Blackboard is a web-based distance learning tool used by universities and other higher education institutions that enables students to continue their studies [7]. It is one of the most widely used web-based platforms and is currently used by all Saudi Arabian universities [8].

Most telecommunications firms in Saudi Arabia (eg, Saudi Telecom Company, Mobily, and Zain Saudi Arabia) aided in efficiently delivering distance learning and health care during the pandemic. These companies offered free data services to the most widely used telehealth and health applications, as well as educational platforms [3]. In addition, the Saudi Ministry of Education introduced distance learning programs to safely and effectively continue the educational process. Within days, all universities, including medical schools, switched to distance mode of education delivery. Medical schools altered their teaching approach as a result of this tremendous, unforeseen shift from traditional learning to an exclusively distance learning environment [9].

The components of distance learning delivery include ease of information access, material distribution, updating, and standardization. Distance learning allows for rapid and easy content revision to achieve learning objectives [10]. Furthermore, it provides several opportunities for students to improve their autonomous learning and enables students to access learning through distance learning tools and materials anytime and anywhere. Moreover, distance education provides excellent opportunities for students to enhance their connection with instructors [11]. Nevertheless, the appropriate conditions are necessary for the creation of a productive learning atmosphere. In addition, the development of technical infrastructure, the purchase and maintenance of tools and equipment, the instruction of human resources, and the production of distance content are costly and time-consuming processes. The institution, administrators, teachers, and students require information technology resources and skills. Poor time management and a lack of self-control have been reported as the 2 major factors affecting students’ perception of distance learning [12].

A lack of in-person, face-to-face interactions in the classroom or workplace characterizes distance learning classes. Interpersonal communication is vital for numerous students and organizations. For instance, in-person interaction with lecturers and group discussions are crucial motivational activities and learning techniques [6]. Team-based learning (TBL) is a small-group, active learning method that incorporates rapid feedback, teamwork, and solo work to help students apply conceptual knowledge [13]. These activities significantly affect students’ class behavior and comprehension of lectures. However, the effectiveness of distance learning courses depends on individual factors, such as the student’s home setting, socioeconomic factors, and the educational level of their parents [6].

Most universities effectively transitioned their teaching operations from traditional to distance learning in response to the pandemic. However, several problems emerged, including poor teaching quality, work overload, lack of accessibility, student nervousness and dissatisfaction, fundamental questions concerning the use of distance education in the future, the requirement for suitable educational staff support and education to handle the inescapable adjustments required for implementing contemporary educational methods in a distance learning setting, and appropriate teacher preparation. Students expressed concern about the ability of distance education to replace human interaction in terms of emotion [14].

COVID-19 affected the reception and delivery of education at all levels [15], with educational authorities in most countries implementing distance learning strategies [11]. However, the sudden conversion to distance learning affected the performance of students and teachers in all learning environments, potentially influencing the efficacy of distance learning. Many academic staff members had no previous experience teaching using distance learning. Students may have been more anxious than usual due to worries regarding infection, timely graduation, finances, and employment, which could have had a detrimental effect on their academic achievement [16]. Synchronized technology enables teachers and pupils to interact “live.” Particularly under uncertain global conditions, such as pandemics, distance learning can result in more efficient and straightforward access to knowledge [9]. Distance education can also serve as a tool to mitigate social isolation and assist in outbreak control. It also provides a flexibility that allows for learning regardless of location or time, which is essential for education during periods of crisis. In addition, the success of the technical infrastructure at schools and colleges depends on adequate maintenance [17]. This study aims to assess pharmacy students’ perceptions of distance learning and to evaluate the postpandemic distance learning environment. We also try to identify the factors affecting distance learning and to assess the emotions experienced by students during their period of distance education.

## Methods

### Study Design and Setting

A web-based survey was used to conduct this cross-sectional study. This study’s intended participants were students enrolled in the doctor of pharmacy (PharmD) program at Unaizah College of Pharmacy, Qassim University, Saudi Arabia, during the 2022-2023 academic year. PharmD students and interns who completed 1 year of distance learning during the COVID-19 pandemic using distance learning platforms provided by Qassim University were eligible for this study. The research did not include students who were attending other colleges. In addition, we excluded students who had not completed 1 year of distance learning at the university, similar to first-year students in the PharmD program, since they were not enrolled in the university during the COVID-19 pandemic.

### Sample Size

The target population in this study comprised male and female third-year to sixth-year (internship year) PharmD students from Unaizah College of Pharmacy, Qassim University. According to the college’s students’ academic affairs office, the number of enrolled students was 327. Raosoft (Sample Size Calculator; Raosoft, Inc) was used to compute the sample size. With a 50% response rate, a 5% margin of error, and a CI level of 95%, the sample size should not be fewer than 141 students [18].

### Study Tool

The questionnaire used in this study was created based on a prior study by Kedraka et al [14], and the authors obtained permission to use the questionnaire. A survey composed of multiple-choice questions was created using a web-based survey creator. That survey consisted of 34 questions assessing attitudes toward distance learning [14]. We slightly modified some questions to comply with the teaching methods used at Unaizah College of Pharmacy. Subject experts validated this modified questionnaire for clarity, understandability, and applicability. The final survey had 6 sections. The first section included 5 questions to assess the necessary abilities of students in meeting learning objectives and measuring progress. Student preferences also play a crucial role in the learning process. The second section comprised 9 questions that could serve as recommendations for developing a distance learning environment and measuring student performance in distance classrooms. Performance is greatly influenced by technical knowledge, comfort level, and attitudes toward using the internet for learning. Using a 5-point scale (1: not at all to 5: very much), we evaluated the opinions of the participants regarding the effectiveness of remote learning in terms of meeting the needs of the course, satisfaction with communication with the instructor, level of interest in the new method of teaching, level of course participation, interactions between students and teachers, and interactions between students. These factors may provide insight into the perceptions of students regarding the distance learning environment.

The third section, which included 4 questions, aimed to assess remote learning quality by identifying influential factors and motivating elements. The fourth section included 4 questions used to assess successful communication between students and teachers in distance courses, recommend steps to enhance interaction, and lessen the isolation associated with distance courses. The fifth section consisted of 6 questions intended to identify elements lost due to the shift from face-to-face interaction to distance learning, improve course delivery, and understand the factors that constitute a successful distance learning experience. Distance learning requires a critical understanding of the effects of interactions on student engagement. The sixth section consisted of 6 questions focusing on the motives and sentiments of the students toward their participation in the educational program. All eligible students were asked to participate in this study, and the questionnaire was disseminated web-based using WhatsApp (WhatsApp LLC).

### Ethical Considerations

The ethics permission for this study was granted by Qassim University’s Health Research Ethics Committee in Saudi Arabia (22-17-11). The participation in this study was voluntary and informed consent to take part in this study was obtained at the beginning of the survey and the statements on the anonymity of responses or confidentiality of data were maintained throughout this study. No compensation or reward was offered to this study’s participants.

### Statistical Analysis

The data were analyzed and student replies were compiled using SPSS (version 20.0; IBM Corp). The survey results were compiled using descriptive statistics comprising frequencies and percentages. Further reported were the mean scores for male and female students.

## Results

### Demographic Data

The survey was completed by 141 students (female: n=58, 41%; male: n=83, 58.9%) from the Unaizah College of Pharmacy. The greatest number of participants were recruited from the fifth year (51/141, 36.2%), followed by the third year (36/141, 25.5%), fourth year (30/141, 21.3 %), and sixth year (24/141, 17%).

### Preference for Continuing Distance Education After the Pandemic

As shown in Table 1, most of the students (102/141, 72.3%) were unwilling to continue distance education (male: 50/83, 60.2%; female: 52/58, 89.6%; not at all or a little) after the pandemic for courses that included laboratory activities. In contrast, the majority of the students, 68% (96/141), had a favorable view regarding the continuation of distance education for elective courses (male: 53/83, 63.8%; female: 43/58, 74.1%; much or very much). Regarding TBL examinations, the majority of students, 60.3% (85/141), did not wish to continue taking distance examinations after the pandemic (male: 38/83, 45.8%; female: 47/58, 81%).

Regarding lectures, less than half of the students (63/141, 44.6%) did not wish to continue taking the lectures via distance platforms (male: 36/83, 43.4%; female: 27/58, 46.5%). Approximately 46.8% (66/141) of students wished to continue receiving their education distance for seminars (male: 41/83, 49.4%; female: 25/58, 43.1%).

**Table 1:** Pharmacy students’ preference for continuing distance education after the pandemic.

Type of course and sex			Not at all, n (%)		A little, n (%)		Quite, n (%)		Much, n (%)		Very much, n (%)		Mean (SD)
**Lectures**													
	Male (n=83)	20 (24)		16 (19.3)		11 (13.3)		16 (19.3)		20 (24.1)		3.0 (1.5)	
	Female (n=58)	10 (17.2)		17 (29.3)		8 (13.9)		14 (24.1)		9 (15.5)		2.9 (1.4)	
**Elective courses**													
	Male (n=83)	11 (13.3)		7 (8.4)		12 (14.4)		13 (15.7)		40 (48.2)		3.8 (1.5)	
	Female (n=58)	4 (6.9)		3 (5.2)		8 (13.8)		14 (24.1)		29 (50)		4.1 (1.2)	
**Laboratory courses**													
	Male (n=83)	41 (49.4)		9 (10.8)		8 (9.7)		6 (7.2)		19 (22.9)		2.4 (1.7)	
	Female (n=58)	40 (69)		12 (20.7)		2 (3.4)		2 (3.4)		2 (3.4)		1.5 (1.0)	
**Team-based learning**													
	Male (n=83)	28 (33.7)		10 (12)		13 (15.7)		14 (16.9)		18 (21.7)		2.8 (1.6)	
	Female (n=58)	33 (56.9)		14 (24.1)		4 (7)		2 (3.4)		5 (8.6)		1.8 (1.2)	
**Seminars**													
	Male (n=83)	19 (22.9)		6 (7.2)		17 (20.5)		6 (7.2)		35 (42.2)		3 (1.6)	
	Female (n=58)	20 (34.5)		8 (13.8)		5 (8.6)		12 (20.7)		13 (22.4)		2.8 (1.6)	

### Assessment of the Distance Educational Environment

As shown in Table 2, most participants had positive opinions regarding the former’s educational potential of the distance versus the conventional learning environment. Male students exhibited a more favorable attitude (higher average) compared with female students in all statements that assessed the distance learning environment compared to conventional learning on campus. Using a 5-point scale (1: not at all to 5: very much), we evaluated the opinions of the participants regarding the effectiveness of remote learning in terms of meeting the needs of the course (male: 3.5; female: 2.9), satisfaction with communication with the instructor (male: 3.6; female: 3.1), level of interest in the new method of teaching (male: 3.5; female: 2.6), level of course participation (male: 3; female: 2.9), interactions between students and teachers (male: 3.2; female: 2.7), and interactions between students (male: 3; female: 2.8).

Less than half of the students (61/141, 43.3%; male: 39/83, 47%; female: 22/58, 37.9%) believed that the course content was understandable. Additionally, 83.7% (118/141) of the students indicated that taking the course online was simple (male: 70/83, 84.3%; female: 48/58, 82.7%; much or very much). As for developing new skills, 38.3% (54/141) of the students thought that they developed new skills related to distance education (male: 40/83, 48.2%; female: 14/58, 24%).

**Table 2:** Pharmacy students’ attitudes about the remote learning environment.

Assessment and sex			Not at all, n (%)		A little, n (%)		Quite, n (%)		Much, n (%)		Very much, n (%)		Mean (SD)
**The content** **of the course is comprehensible**													
	Male (n=83)	5 (6)		7 (8.4)		32 (38.6)		20 (24.1)		19 (22.9)		3.5 (1.1)	
	Female (n=58)	6 (10.3)		11 (19)		19 (32.8)		14 (24.1)		8 (13.8)		3.1 (1.2)	
**New skills related to distance education are being developed**													
	Male (n=83)	6 (7.2)		12 (14.5)		25 (30.1)		23 (27.7)		17 (20.5)		3.4 (1.2)	
	Female (n=58)	13 (22.4)		11 (19)		20 (34.5)		12 (20.7)		2 (3.4)		2.6 (1.2)	
**The teaching** **method of distance learning covers the prerequisites of the course**													
	Male (n=83)	10 (12)		7 (8.4)		19 (23)		26 (31.3)		21 (25.3)		3.5 (1.3)	
	Female (n=58)	13 (22.4)		11 (19)		9 (15.5)		18 (31)		7 (12.1)		2.9 (1.4)	
**The new** **mode of teaching is interesting**													
	Male (n=83)	8 (9.6)		9 (10.8)		20 (24.2)		24 (28.9)		22 (26.5)		3.5 (1.3)	
	Female (n=58)	16 (27.6)		10 (17.2)		15 (25.9)		14 (24.1)		3 (5.2)		2.6 (1.3)	
**Communication with the teacher is satisfactory**													
	Male (n=83)	6 (7.2)		11 (13.3)		18 (21.7)		22 (26.5)		26 (31.3)		3.6 (1.3)	
	Female (n=58)	9 (15.5)		13 (22.4)		8 (13.8)		19 (32.8)		9 (15.5)		3.1 (1.3)	
**Participation in class is great**													
	Male (n=83)	8 (9.6)		16 (19.3)		18 (21.7)		18 (21.7)		23 (27.7)		3.4 (1.3)	
	Female (n=58)	13 (22.4)		13 (22.4)		10 (17.3)		13 (22.4)		9 (15.5)		2.9 (1.4)	
**Attendance** **is easy**													
	Male (n=83)	5 (6)		3 (3.6)		5 (6.1)		19 (22.9)		51 (61.4)		4.3 (1.1)	
	Female (n=58)	1 (1.7)		3 (5.2)		6 (10.4)		18 (31)		30 (51.7)		4.3 (1.0)	
**Interaction** **between teacher and students is great**													
	Male (n=83)	13 (15.7)		9 (10.8)		29 (34.9)		13 (15.7)		19 (22.9)		3.2 (1.3)	
	Female (n=58)	16 (27.6)		10 (17.2)		16 (27.6)		9 (15.5)		7 (12.1)		2.7 (1.4)	
**Interaction** **among students is great**													
	Male (n=83)	17 (20.5)		9 (10.8)		10 (12.1)		19 (22.9)		28 (33.7)		3.4 (1.5)	
	Female (n=58)	13 (22.4)		13 (22.4)		12 (20.7)		13 (22.4)		7 (12.1)		2.8(1.3)	

### Factors Affecting e-Learning

As shown in Table 3, more than half of the participants (79/141, 56%) reported that having the camera on during class impacted their learning (male: 49/83, 59%; female: 30/58, 51.7%). Further, only 29.1% (41/141) of the students indicated that nonvisual communication with their fellow students would impact their learning (male: 22/83, 26.5%; female: 19/58, 32.7%). About 29.7% (42/141) of the students reported that the teacher’s insufficient knowledge concerning the handling of the platform impacted distance learning (male: 30/83, 36.1%; female: 12/58, 21%), while 44.6% (63/141) indicated that the inability to cooperate with their fellow students would affect distance learning (male: 44/83, 53%; female: 19/58, 32.7%).

**Table 3:** Factors affecting e-learning.

Factors and sex			Not at all, n (%)		A little, n (%)		Quite, n (%)		Much, n (%)		Very much, n (%)		Mean (SD)
**The teacher’s camera should be turned on**													
	Male (n=83)	12 (14.5)		9 (10.8)		13 (15.7)		18 (21.7)		31 (37.3)		3.56 (1.4)	
	Female (n=58)	1 (1.7)		12 (20.7)		15 (25.9)		18 (31)		12 (20.7)		3.48 (1.1)	
**Teacher’s deficiencies in knowledge of handling the platform**													
	Male (n=83)	15 (18.1)		11 (13.3)		27 (32.4)		14 (16.9)		16 (19.3)		3.06 (1.3)	
	Female (n=58)	8 (13.8)		20 (34.5)		18 (31)		7 (12.1)		5 (8.6)		2.67 (1.1)	
**Nonvisual communication with my fellow students**													
	Male (n=83)	24 (28.9)		12 (14.5)		25 (30.1)		9 (10.8)		13 (15.7)		2.69 (1.4)	
	Female (n=58)	10 (17.2)		18 (31)		11 (19.1)		9 (15.5)		10 (17.2)		2.84 (1.4)	
**Noncooperation with my fellow students**													
	Male (n=83)	9 (10.8)		9 (10.8)		21 (25.4)		22 (26.5)		22 (26.5)		3.46 (1.3)	
	Female (n=58)	17 (29.3)		9 (15.5)		13 (22.4)		12 (20.7)		7 (12.1)		2.70 (1.4)	

### Preferred Modes of Communication With Instructors

As shown in Table 4, the participants’ preferred method of communication was the course chat (96/141, 68%; male: 59/83, 71.1%; female: 37/58, 63.8%). The second preferred method of communication for male students was a microphone (47/83, 56.6%), followed by face-to-face communication in the classroom (47/83, 56.6%) and groups on Zoom (37/83, 44.6%) throughout the course. The second preferred method of communication for female students was face-to-face communication in the classroom (36/58, 62%), followed by groups on Blackboard or Zoom (26/58, 44.8%). The least preferred option was a microphone (25/58, 43.1%).

**Table 4:** Pharmacy students’ preferred mode of communication with the instructor.

Ways of communication and sex		Not at all, n (%)	A little, n (%)	Quite, n (%)	Much, n (%)	Very much, n (%)	Mean (SD)
**On chat**							
	Male (n=83)	5 (6)	5 (6)	14 (16.9)	26 (31.3)	33 (39.8)	3.9 (1.2)
	Female (n=58)	9 (15.5)	8 (13.8)	4 (6.9)	22 (37.9)	15 (25.9)	3.4 (1.4)
**Speaking on the internet**							
	Male (n=83)	6 (7.2)	9 (10.8)	21 (25.4)	22 (26.5)	25 (30.1)	3.6 (1.2)
	Female (n=58)	8 (13.8)	9 (15.5)	16 (27.6)	17 (29.3)	8 (13.8)	3.1 (1.2)
**Internet-based room**							
	Male (n=83)	8 (9.6)	12 (14.5)	26 (31.3)	14 (16.9)	23 (27.7)	3.4 (1.3)
	Female (n=58)	8 (13.8)	8 (13.8)	16 (27.6)	17 (29.3)	9 (15.5)	3.2 (1.3)
**In the classroom**							
	Male (n=83)	9 (10.8)	9 (10.8)	18 (21.8)	22 (26.5)	25 (30.1)	3.5 (1.3)
	Female (n=58)	4 (6.9)	9 (15.5)	9 (15.6)	22 (37.9)	14 (24.1)	3.6 (1.2)

### Traditional Education Components not Present in Distance Learning

As shown in Table 5, a large proportion of students, 58.9% (83/141), reported impaired social engagement around the campus (male: 45/83, 54.2%; female: 38/58, 65.5%), while 45.4% (64/141) of the students reported that they missed their classmates (male: 36/83, 43%; female: 28/58, 48.3%), in-person interactions during classes (62/141, 44%; male: 33/83, 39.8%; female: 29/58, 50%), and interactions in the classroom (60/141, 42.5%; male: 31/83, 37.3%; female: 29/58, 50%). Only 31.2% (44/141) of students stated that they missed their professors (male: 27/83, 32.5%; female: 17/58, 29.3%), while 37.5% (53/141) of the students stated that they missed being able to visit the library (male: 21/83, 25.3%; female: 32/58, 55.2%).

**Table 5:** Components of traditional learning that are currently missing from remote education.

Element and sex		Not at all, n (%)	A little, n (%)	Quite, n (%)	Much, n (%)	Very much, n (%)	Mean (SD)
**Educators**							
	Male (n=83)	14 (16.9)	11 (13.3)	31 (37.3)	17 (20.5)	10 (12)	3.0 (1.2)
	Female (n=58)	9 (15.5)	19 (32.8)	13 (22.4)	15 (25.9)	2 (3.4)	2.7 (1.1)
**Fellow students**							
	Male (n=83)	10 (12)	7 (8.4)	30 (36.2)	16 (19.3)	20 (24.1)	3.3 (1.3)
	Female (n=58)	6 (10.3)	16 (27.6)	8 (13.8)	15 (25.9)	13 (22.4)	3.2 (1.4)
**Library**							
	Male (n=83)	30 (36.1)	10 (12)	22 (26.6)	11 (13.3)	10 (12)	2.5 (1.4)
	Female (n=58)	7 (12.1)	10 (17.2)	9 (15.5)	15 (25.9)	17 (29.3)	3.4 (1.4)
**Classrooms**							
	Male (n=83)	18 (21.7)	12 (14.5)	22 (26.4)	19 (22.9)	12 (14.5)	2.9 (1.4)
	Female (n=58)	9 (15.5)	11 (19)	9 (15.5)	10 (17.2)	19 (32.8)	3.3 (1.5)
**In-person communication during lessons**							
	Male (n=83)	10 (12)	12 (14.5)	28 (33.7)	17 (20.5)	16 (19.3)	3.2 (1.3)
	Female (n=58)	10 (17.2)	5 (8.6)	14 (24.2)	14 (24.1)	15 (25.9)	3.3 (1.4)
**Social interaction**							
	Male (n=83)	9 (10.8)	4 (4.8)	25 (30.2)	22 (26.5)	23 (27.7)	3.6 (1.3)
	Female (n=58)	4 (6.9)	7 (12.1)	9 (15.5)	17 (29.3)	21 (36.2)	3.8 (1.3)

### Emotions Experienced During Distance Education

As shown in Table 6, a significant majority of the students, 73.7% (104/141), expressed relief that their classes had not been disrupted (male: 59/83, 71.1%; female: 45/58, 77.6%). However, there was still much interest (74/141, 52.5%) in how the studies would go (male: 50/83, 60.2%; female: 24/58, 41.3%), and much enthusiasm among students for their first distance learning experiences (82/141, 58.1%; male: 50/83, 60.2%; female: 32/58, 55.2%). Additionally, the majority of students, 80.1% (113/141; male: 67/83, 80.7%; female: 46/58, 79.3%), liked not having to go to their lessons, whereas only 40.4% (57/141) of students were satisfied that the distance courses would continue (male: 34/83, 40.9%; female: 23/58, 39.6%).

**Table 6:** Emotions experienced by students during distance education.

Emotion and sex			Not at all, n (%)		A little, n (%)		Quite, n (%)		Much, n (%)		Very much, n (%)		Mean (SD)
**Joy at classes not being held**													
	Male (n=83)	14.0 (16.9)		9.0 (10.8)		26.0 (31.3)		19.0 (22.9)		15.0 (18.1)		3.1 (1.3)	
	Female (n=58)	9.0 (15.5)		9.0 (15.5)		17.0 (29.3)		12.0 (20.7)		11.0 (19)		3.1 (1.3)	
**Pleasure at not having to commute to attend classes**													
	Male (n=83)	2.0 (2.4)		3.0 (3.6)		11.0 (13.3)		17.0 (20.5)		50.0 (60.2)		4.3 (1.0)	
	Female (n=58)	2.0 (3.4)		5.0 (8.6)		5.0 (8.7)		12.0 (20.7)		34.0 (58.6)		4.2 (1.1)	
**Relief at not losing the semester**													
	Male (n=83)	1.0 (1.2)		5.0 (6)		18.0 (21.7)		21.0 (25.3)		38.0 (45.8)		4.1 (1.0)	
	Female (n=58)	2.0 (3.4)		4.0 (6.9)		7.0 (12.2)		14.0 (24.1)		31.0 (53.4)		4.2 (1.1)	
**Enthusiasm for the new experience**													
	Male (n=83)	7.0 (8.4)		6.0 (7.2)		20.0 (24.2)		19.0 (22.9)		31.0 (37.3)		3.7 (1.3)	
	Female (n=58)	8.0 (13.8)		11.0 (19)		7.0 (12)		12.0 (20.7)		20.0 (34.5)		3.4 (1.5)	
**Disappointment because the new educational environment does not work for me**													
	Male (n=83)	15.0 (18.1)		6.0 (7.2)		30.0 (36.1)		15.0 (18.1)		17.0 (20.5)		3.2 (1.3)	
	Female (n=58)	9.0 (15.5)		9.0 (15.5)		16.0 (27.6)		15.0 (25.9)		9.0 (15.5)		3.1 (1.3)	
**Curiosity regarding the mood of studies in the future**													
	Male (n=83)	1.0 (1.2)		5.0 (6)		27.0 (32.5)		32.0 (38.6)		18.0 (21.7)		3.7 (0.9)	
	Female (n=58)	5.0 (8.6)		7.0 (12.1)		22.0 (37.9)		13.0 (22.4)		11.0 (19)		3.3 (1.2)	

## Discussion

### Principal Findings

In this study, we attempted to comprehend student opinions, attitudes, and worries regarding distance learning and the barriers and difficulties they faced. Data were gathered through a web-based survey questionnaire between December 2022 and January 2023. The results of our study demonstrate that the participants showed good attitudes to the continued delivery of lectures and elective courses via distance learning. These findings support previous research showing that 63% of students preferred distance learning for the lectures compared to 29% for offline lectures [19]. In contrast, most had negative attitudes toward distance learning for laboratory courses and TBL examinations. These findings support previous research showing that students had negative perceptions of distance learning in many ways of teaching. According to Kedraka et al [14], most of the students at 2 participating universities (University of Patras: 84.1%; Democritus University of Thrace: 98%) did not wish to continue their education for laboratory courses distance beyond the epidemic. A study conducted by Stoian et al [20] reported that 38.8% preferred using face-to-face education, which seemed the most beneficial for their professional development, followed by a distance education rate of 34.3%, then both (face-to-face and distance education) at 27%.

Students’ perceptions of the distance learning environment were positive, with 83.7% (118/141) of the students stating that taking a course through distance learning was easy and 43.3% (61/141) finding the course content understandable. Our findings are in agreement with Bani Hani et al [10], who revealed that more than half of the students participating in the study (615/999) stated they gained the same or even better knowledge than what they gained before the pandemic and around half (458/999) of all the students recognize the university’s e-learning website as available for easy access. A Saudi study [21] showed that 49.2% of students exhibited positive attitudes toward the provided distance learning, and 34% of students identified some barriers to the provision of distance learning.

The majority of participants, 56% (79/141), felt that having a camera on during class had a detrimental influence on their learning. In a survey conducted by Gherheş et al [22], more than half of students claimed that they were unwilling to have their cameras on during distance sessions. Furthermore, 76.5% of participants stated that they did not use webcams when communicating via social media [23]. In contrast, in a study performed by Pullan et al [24], 51% of participants reported that having a camera turned on did not change their ability to engage, while 38% thought that having a camera on improved their engagement.

More than half of the students, 58.9% (83/141), claimed to have impaired social contacts on and around campus, and 44% (62/141) reported having impaired in-person interactions with teachers during class, with 42.5% (60/141) reporting poor interactions in the classroom. These findings support previous research. Muthuprasad et al [25] reported that 60% of respondents agreed with the assertion that contact with the instructor is less effective in distance classrooms as compared to face-to-face classes. Another study [26] revealed that a lack of live communication reduces the effectiveness of distance learning (52.1%). Furthermore, a study by Dodd et al [27] revealed that 84.6% of the students found it difficult to interact with other students, teachers 74.6%, and 74.7% reported more difficulty in distance learning than face-to-face.

The preferred method of communication (96/141, 68%) for the students in our study was the course chat, a result that supports earlier studies. Coman et al [28] found that 52.4% of students preferred to communicate with teachers through writing on chat. A study [29] revealed that most students (87.4%) preferred synchronized learning sessions for group discussions. In addition, 61.7% of students disagreed that distance learning provides similar learning satisfaction to classroom learning.

As for feelings, 73.7% (104/141) of students were relieved that their courses had not been interrupted and 58.1% (82/141) had enthusiasm for the new experience. However, there was still much interest, 52.5% (74/141), in how the studies would go in the future. Murphy et al [30] reported that their participants expressed uncertainty (59.5%), anxiety (50.7%), and nervousness (41.2%) regarding the switch to distance courses. In addition, Guse et al [31] revealed in a survey conducted in May 2020 on students that 45.5% of dental students noticed a decline in their excitement to study compared to 30.7% of medical students. Worsening mental health was reported by 36.4% (16/44) and 29.5% (26/88) of dental and medical students, respectively.

As this study was conducted in a single institution, its conclusions may not apply to other Saudi Arabian or global academic institutions. The findings of this study may be used to design a more effective and user-friendly distance course. As the study design is cross-sectional, the results only reflect the situation of distance learning during the data collection.

### Conclusions

Similar to all types of education, distance learning is, as reported by the students, characterized by advantages and disadvantages, an understanding of which will aid educational institutions in developing plans for the more effective distribution of instructional materials to students. According to the results, the students felt that the course material was intelligible and that the distance courses were uncomplicated. Moreover, they expressed relief that their studies were not disrupted. These benefits facilitate the design of courses that meet the requirements of students and may lead to advancements in distance learning techniques.

Students in this study identified loss of face-to-face contact during their courses as the greatest drawback of remote education, followed by a lack of social connection on campus. This lack of peer interaction is particularly notable, for it is crucial to understand the importance of peer connection for students. Therefore, these variables should be considered for the improvement of e-learning. In summary, face-to-face communication is an indispensable part of the educational process.

## Abbreviations

TBL: team-based learning

## Data Availability

The data sets used for this study are available upon reasonable request.
